# Eocene *Podocarpium* (Leguminosae) from South China and its biogeographic implications

**DOI:** 10.3389/fpls.2015.00938

**Published:** 2015-11-03

**Authors:** Qingqing Xu, Jue Qiu, Zhekun Zhou, Jianhua Jin

**Affiliations:** ^1^State Key Laboratory of Biocontrol and Guangdong Provincial Key Laboratory of Plant Resources, School of Life Sciences, Sun Yat-sen UniversityGuangzhou, China; ^2^Department of Paleobiology, National Museum of Natural History, Smithsonian InstitutionWashington, DC, USA; ^3^Xishuangbanna Tropical Botanical Garden, Chinese Academy of SciencesMenglun, China

**Keywords:** Eocene, Leguminosae, *Podocarpium*, phytogeography, South China

## Abstract

*Podocarpium* A. Braun ex Stizenberger is one of the most common legumes in the Neogene of Eurasia, including fossil fruits, seeds, leaves, and possible flower and pollen grains. This genus is not completely consistent with any extant genera according to gross morphological characters and poorly preserved cuticular structures reported in previous studies. The fossil pods collected from the coal-bearing series of the Changchang Basin of Hainan Island and Maoming Basin of Guangdong, South China, are examined by morphologically comparative work, with special reference to venation patterns and placental position. These distinctive features, as well as the ovule development of pods from different developmental stages and the epidermal structure of the pods, as distinguished from previous records lead to the conclusion that these fossils can be recognized as a new species of *Podocarpium*, *P. eocenicum* sp. nov. This new discovery indicates that *Podocarpium* had arrived in South China by the Eocene. Investigation on the fossil records of this extinct genus shows that *P. eocenicum* is the earliest and lowest latitude fossil data. The possible occurrence pattern of this genus is revealed as follows: *Podocarpium* had distributed in the South China at least in the middle Eocene, and then migrated to Europe during the Oligocene; in the Miocene this genus reached its peak in Eurasia, spreading extensively across subtropical areas to warm temperate areas; finally, *Podocarpium* shrank rapidly and became extinct in Eurasia during the Pliocene.

## Introduction

*Podocarpium* A. Braun ex Stizenberger (formerly *Podogonium* Heer) was described as an extinct genus of the Leguminosae and established based on the fruits, seeds, and leaves from the Miocene of Switzerland and southern Germany (Heer, 1857). The nomenclature, taxonomy, and systematic relationship of this genus has been the subject of a long history of controversy (Herendeen, 1992a,b; Wang, 2008). Herendeen (1992b) clarified the confused nomenclature and determined that the correct genus name is *Podocarpium*. In this paper, we also adopt *Podocarpium* as its legitimate generic name. Heer (1857) described six species of *Podocarpium* and five of them were mainly distinguished by the leaflet shape. However, Heer’s taxonomic treatment was poorly accepted because: (1) considerable continuous morphological variation occurs to the fossil leaflets; (2) the fruits of Heer’s original six species can not be distinguished morphologically (Kirchheimer, 1957; Herendeen, 1992a). The recognition of a single variable species of *Podocarpium* was supported by Kirchheimer (1957), Rüﬄe (1963) and Bůžek (1971). This treatment was held by Herendeen (1992a,b) alike, and he proposed a new combination to replace the illegitimate name *Podogonium knorrii* Heer which is universally known. *Podocarpium podocarpum* (A. Braun) Herendeen is validated to be the correct species name (Herendeen, 1992b), and has been conserved and increasingly used (Wang, 2008). The pod of *Podocarpium*, which has very distinctive features, such as generally tardy dehiscence, elliptical shaped, single-seeded and having a long stipe, was originally illustrated as an unidentified fruit by Knorr (1755), and almost a century hence, it was considered to be related to this genus (Braun, 1845; Herendeen, 1992b). *Podocarpium* has been extensively reported in many fossil floras of Eurasia from the early Oligocene to the Pliocene (Liu et al., 2001b; Wang et al., 2007).

Numerous pod specimens collected in the Eocene strata from the Changchang Basin of Hainan Island and Maoming Basin of Guangdong, South China, are described in this paper. A new species *Podocarpium eocenicum* sp. nov. is established based on its shape, size, stipe, certain placental position, specific venation patterns and distinct cuticular structures. It is the first megafossil finding of *Podocarpium* from South China. This discovery also represents the earliest and lowest latitude recorded among all the fossil record of this genus up to the present. Speculation that this genus originated in the early Paleogene of eastern Asia by Liu et al. (2001b) and Wang et al. (2007) was based on possibly related pollen records. This clear megafossil evidence of *Podocarpium* found from the Eocene of South China plays an indispensable role in the tracing of the geographic history of its evolution.

## Materials and Methods

### Geographical and Stratigraphical Information

**FIGURE 1:**
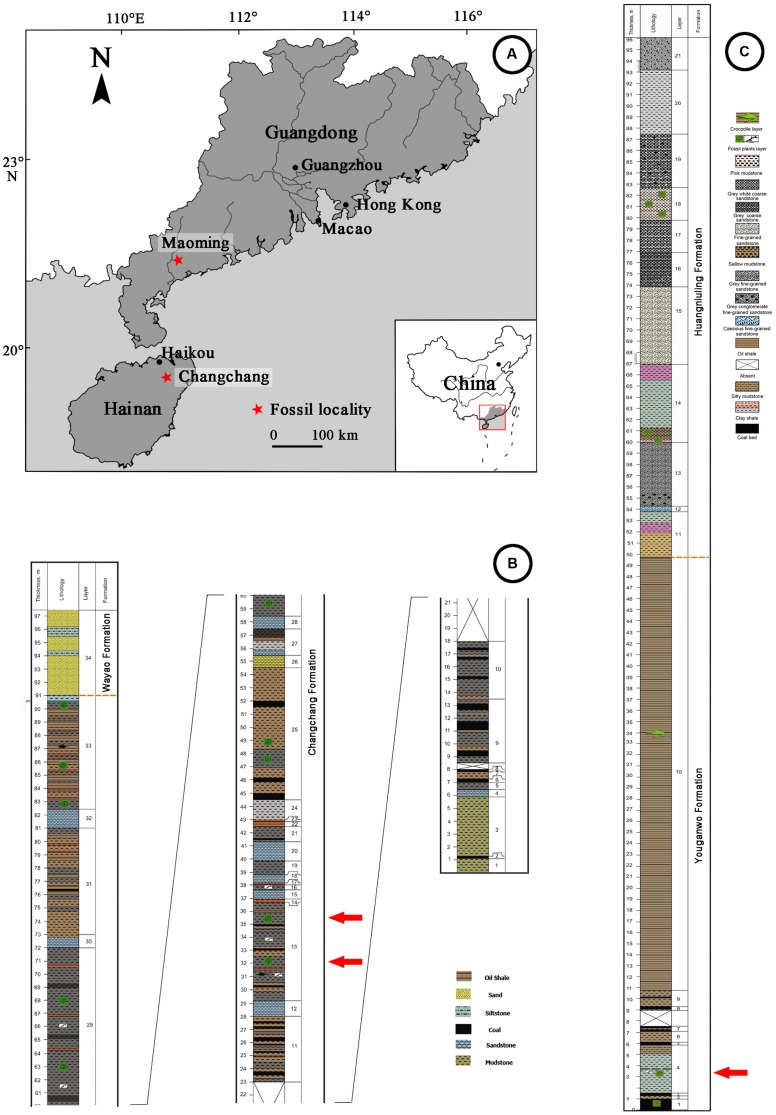
**Geographic map of Changchang Basin, Hainan Island and Maoming Basin, Guangdong Province, and Stratigraphics of fossil localities. (A)** Locations of Changchang Basin and Maoming Basin (red stars), drawn by QQX. **(B)** Lithostratigraphic column of Changchang Basin, modified from Spicer et al. (2014). Specimens were collected from the layers marked by red arrows. **(C)** Lithostratigraphic column of Maoming Basin, modified from Aleksandrova et al. (2012). Specimens were collected from the layer marked by red arrow.

The compressed fossil fruit specimens described in this paper were collected from two coal-bearing fossil sites of South China (**Figure 1A**). Changchang Formation (Fm.) of the Changchang Basin (19°38′N, 110°27′E) is located near Jiazi Town of Qiongshan City, Hainan Island, and Youganwo Fm. of Maoming Basin (21°42′N, 110°53′E) is located near Jintang Town of Maoming City, Guangdong Province.

Changchang Basin, located in the northern part of Hainan Island, can be divided into three formations: Changtou Fm. (Paleocene), and Changchang Fm. and Wayao Fm. (Eocene) (Lei et al., 1992). The Changchang Fm. (**Figure 1B**) is subdivided into the lower part which consists of dark gray mudstone, grayish black coaly shale, brownish gray oil-bearing shale, yellowish brown, grayish yellow, grayish white muddy siltstone and sandstone, and coal. The upper part consists of predominantly lacustrine and fluvial mudstones, siltstones and sandstones. Well preserved plant megafossils were collected mainly from the coal-bearing series of the lower part of the Changchang Fm., including angiosperms (*Castanea* Miller, *Lithocarpus* Blume, *Quercus* L., *Craigia* W. W. Smith et W. E. Evans*, Liquidambar* L., *Myrica* L., *Nelumbo* Adanson, *Paraphyllanthoxylon* Bailey, *Sabalites* Saporta, etc.), gymnosperms (*Nageia* Gaertner, etc.), and ferns (*Osmunda* L., *Salvinia* Séguier, etc.) (Spicer et al., 2014). Based on palynological data and plant assemblages, the Changchang Fm. from which *Podocarpium* was collected is middle Eocene (Lutetian-Bartonian) in age (Spicer et al., 2014).

Maoming Basin is a small inland intramontane basin in southwestern Guangdong Province, China, which is elongated from northwest to southeast. This basin contains one Cretaceous stratum (Tongguling Fm.), and six Palaeo-Neogene strata (given as follows in ascending order: Shangdong Fm., Youganwo Fm., Huangniuling Fm., Shangcun Fm., Laohuling Fm., and Gaopengling Fm.) (Bureau of Geology Mineral Resources of Guangdong Province, 1996). The Youganwo Fm. (**Figure 1C**) is the main mining horizon consisting of the lower brown coal-bearing series and upper dark gray to dark brown densified oil shales. The combustible oil shales enclose the remains of reptilians (e.g., *Anosteira maomingensis* Chow et Liu, *Isomentremys lacuna* Chow et Yeh, *Aspideretes impressus* Yeh, *Adocus inexpectatus* Danilov et al., *Tomistoma petrolica* Yeh, Alligatoridae gen. et sp. indet.), fish (*Cyprinus maomingensis* Liu), and mammals (*Lunania* cf. *L. youngi* Chow) (Aleksandrova et al., 2015). *Podocarpium* was discovered in the lower part of Youganwo Fm. (**Figure 1C**), which is dated as middle Eocene in age on the basis of palynological analysis (Aleksandrova et al., 2015). Associated plant remains include Equisetales, Filicales (Osmundaceae, Polypodiaceae, Salviniaceae), conifers (Podocarpaceae) and numerous angiosperms (Nelumbonaceae, Lauraceae, Fagaceae, Platanaceae, Altingiaceae, Anacardiaceae, Celastraceae, Ulmaceae, Euphorbiaceae, Myrtaceae, etc.) (Aleksandrova et al., 2012, 2015).

### Methods

The fossil specimens of *Podocarpium eocenicum* sp. nov. described in this paper are preserved as impressions and compressions with intact cuticular structures. All the specimens were photographed using a digital camera (Canon Eos 500D). Fossil cuticles were prepared by removing a few fragments from the fossil pods and placing them into deionized (DI) water for 10 min, followed by immersing them with 10% HCl for about 1 h. They were bleached with Schultze’s solution (one part saturated KClO_3_ with two parts 68% HNO_3_) after rinsing with DI water at least three times, and then transferred to 10% NH_3_⋅H_2_O until a dark brown exudate excreted from the fragments. Cuticles were cleaned under a stereoscopic microscope (Leica S8ap0) and then mounted on glass slides using neutral balsam. Slides were observed and photographed using a light microscope (LM) (Nikon-SY100 and ZEISS AXIO Scope.A1) and scanning electron microscope (SEM).

**FIGURE 2:**
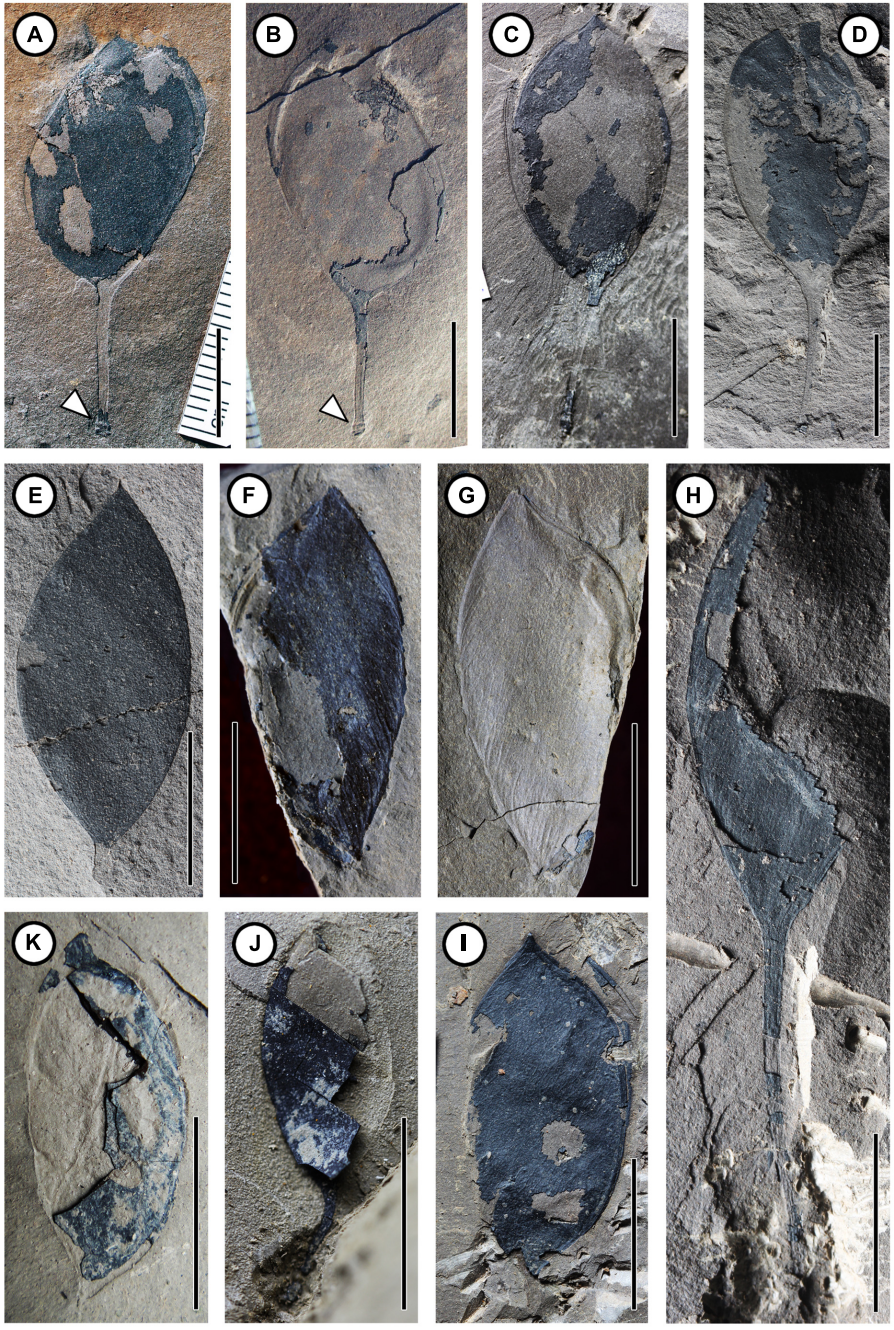
**Fossil pods of *Podocarpium eocenicum* sp. nov. (A)** Dehisced pod with distinct prominent base (arrowhead) in the stipe. CC254a. **(B)** Counterpart of **(A)**. CC254b. **(C)** Dehisced pod. CC1298. **(D–H)** Indehisced pod. **(D)** CC1162b. **(E)** CC1216a. **(F)** Indehisced pod wih distinct venations on the valve. CC1100a. **(G)** Counterpart of **(F)**. CC1100b. **(H)** Incomplete pod with a long stipe and clear venation. CC1217. **(I)** Dehisced pod with venation structures. CC1164. **(J,K)** Indehisced pod. **(J)** MMJ1–002. **(K)** MMJ1–003a. Scale bar = 1 cm.

**FIGURE 3:**
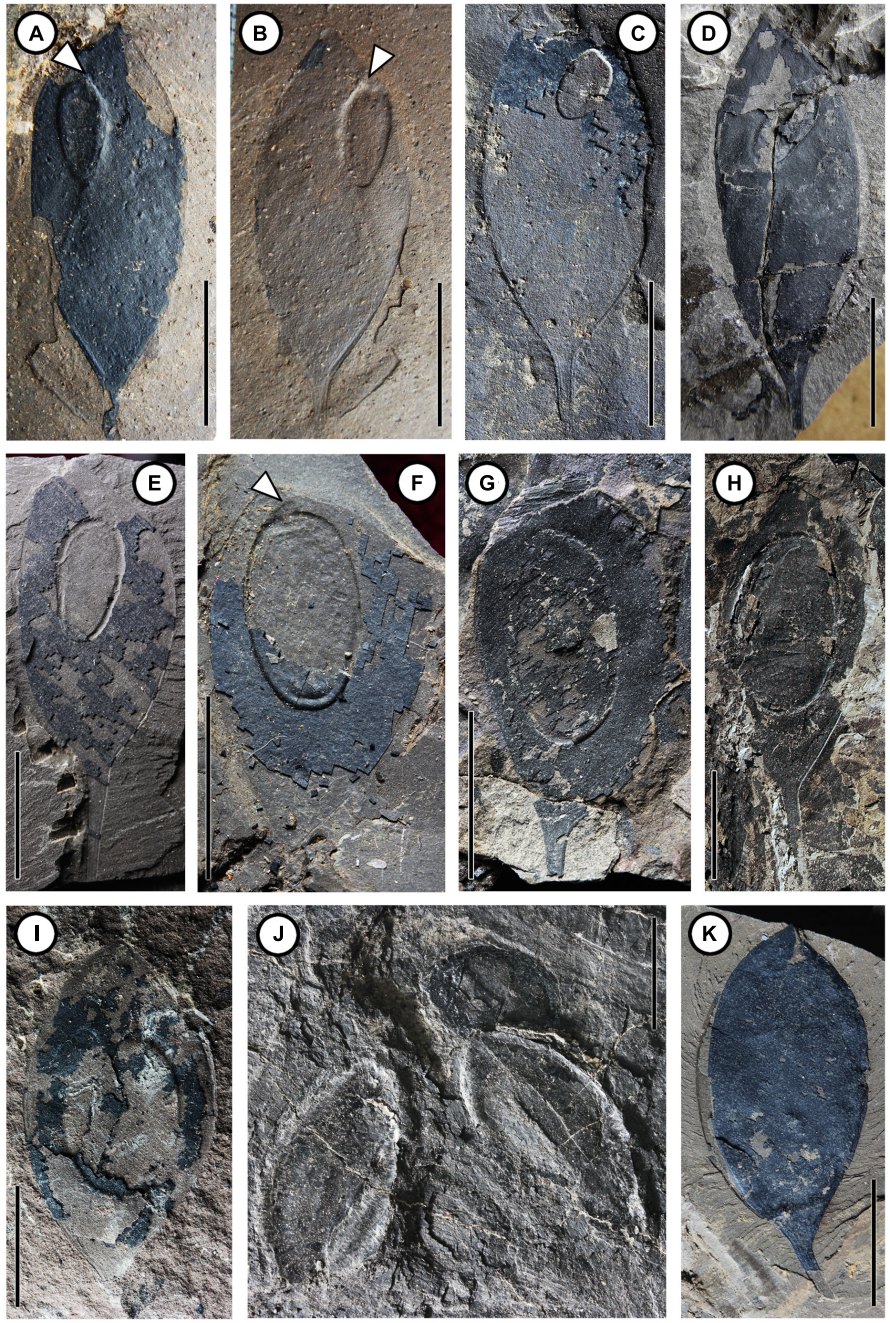
**Different developmental stages of the seed of *Podocarpium eocenicum* sp. nov. (A–E)** Different types of seed located near the apex of the placental suture in the juvenile phase. **(A)** Obovate seed. CC256a. **(B)** Counterpart of **(A)**. CC256b. **(C)** Elliptical seed. CC1168. **(D)** Oblong seed. CC1300. **(E)** Obovate seed. CC1297a. **(F–I)** Seed located near the middle of valve in the maturing phase. **(F)** Elliptical seed with a short funiculus (arrowhead). CC1163a. **(G)** Elliptical to oblong seed situated close to the placental suture. CC1188a. **(H)** Elliptical seed close by the placental suture. CC1184. **(I)** Ellipsoidal seed in the center of the pod. CC698a. **(J)** Seed split out from the valve after maturity. CC1223. **(K)** Dehisced pod without seed. CC1171. Scale bar = 1 cm.

**FIGURE 4:**
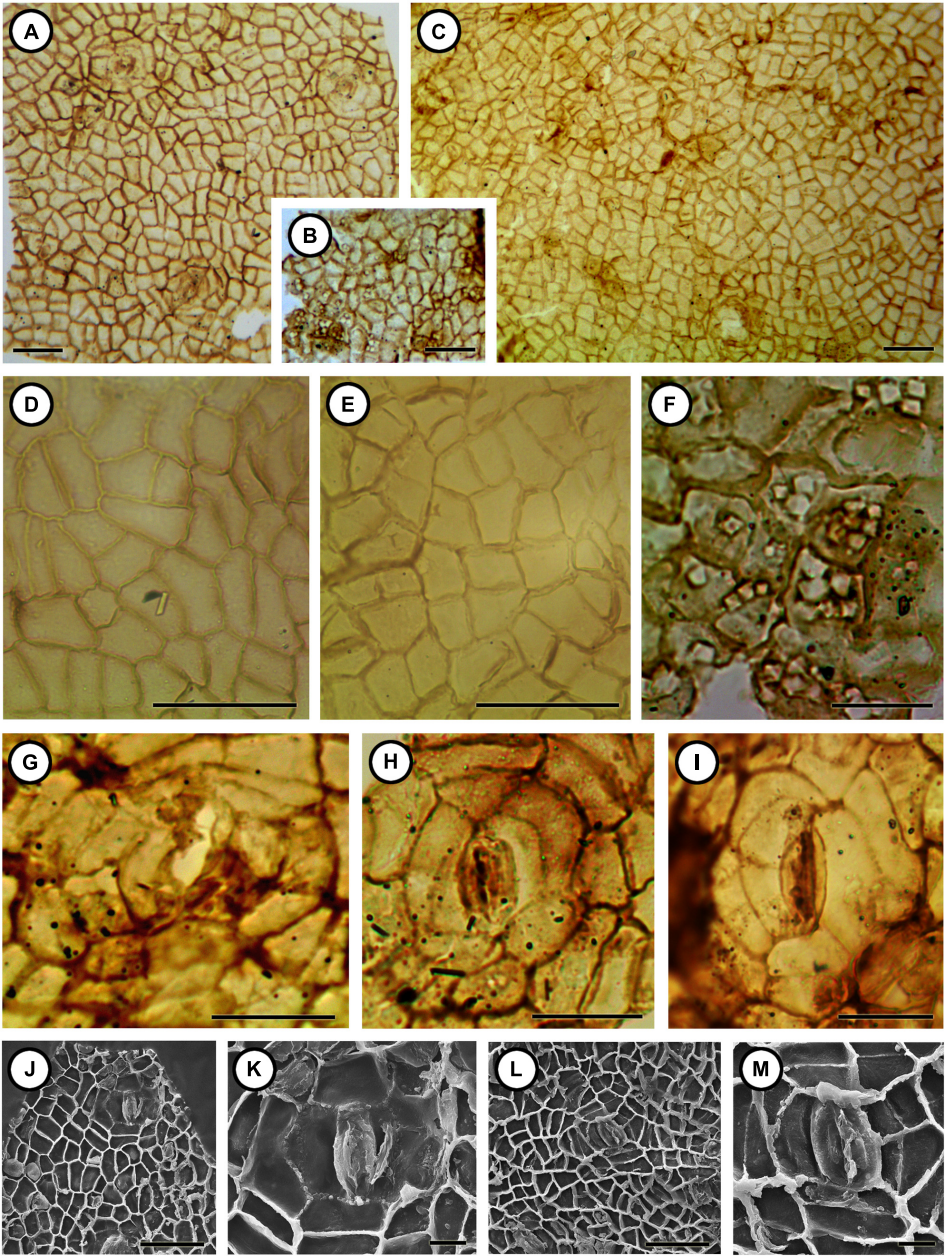
**Cuticular structures of *Podocarpium eocenicum* sp. nov. (A)** Cuticle from the outside of valves shows irregular arrangement of the epidermal cells and stomatal complexes. CC1100a. 10X. Scale bar = 50 μm. **(B)** Cuticle from the inner side of valves shows numerous crystals. CC1100a. 10X. Scale bar = 50 μm. **(C)** Cuticle from the outside of valves shows irregular arrangement of the epidermal cells. MMJ1–003a. 10X. Scale bar = 50 μm. **(D)** Details of the epidermal cells enlarged from **(A)**. CC1100a. 40X. Scale bar = 50 μm. **(E)** Details of the epidermal cells enlarged from (C). MMJ1–003a. 40X. Scale bar = 50 μm. **(F)** Details of the epidermal cells and crystals enlarged from **(B)**. CC1100a. 60X. Scale bar = 25 μm. **(G)** An open stomatal complex with clear outer ledges. CC1300. 60X. Scale bar = 25 μm. **(H)** A closed stomatal complex with seven subsidiary cells. CC1100a. 60X. Scale bar = 25 μm. **(I)** A closed stomatal complex with clear outer ledges. CC1222b. 60X. Scale bar = 25 μm. **(J)** The inner side of cuticle shows irregular arranged epidermal cells and stomatal complex; some cells have patty-like ornamentation. CC1100a. 500X. Scale bar = 50 μm. **(K)** Details of the inner side of a stomatal complex enlarged from **(J)**. CC1100a. 2000X. Scale bar = 10 μm. **(L)** The inner side of cuticle shows irregularly arranged epidermal cells with unevenly thickened anticlinal walls. CC1175. 500X. Scale bar = 50 μm. **(M)** Details of the inner side of the stomatal complex enlarged from **(L)**. CC1175. 2000X. Scale bar = 10 μm.

**FIGURE 5:**
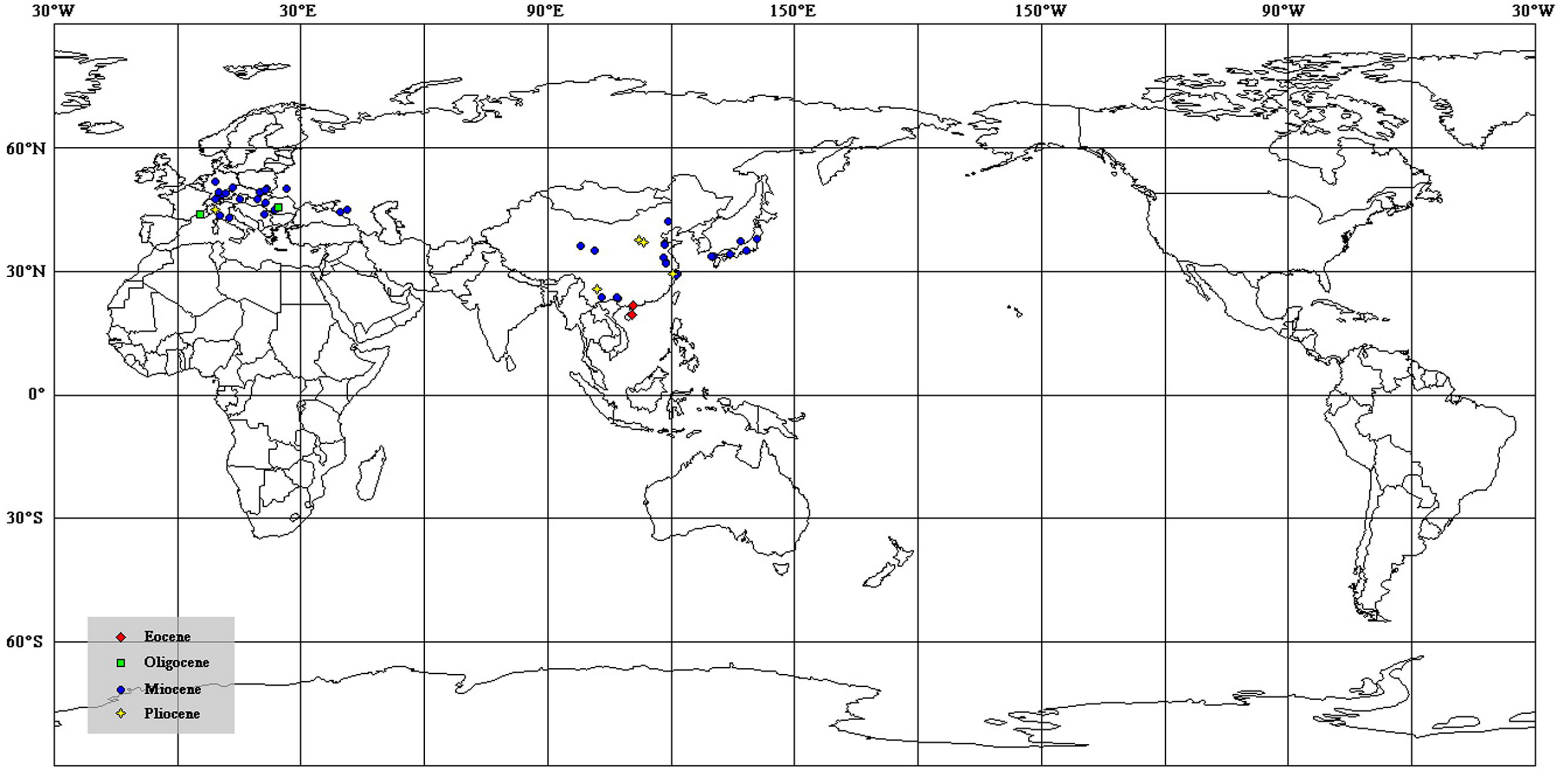
**The distribution of megafossil records of *Podocarpium* on the modern world map [data from Liu et al. (2001b) and Wang et al. (2007)]**.

**FIGURE 6:**
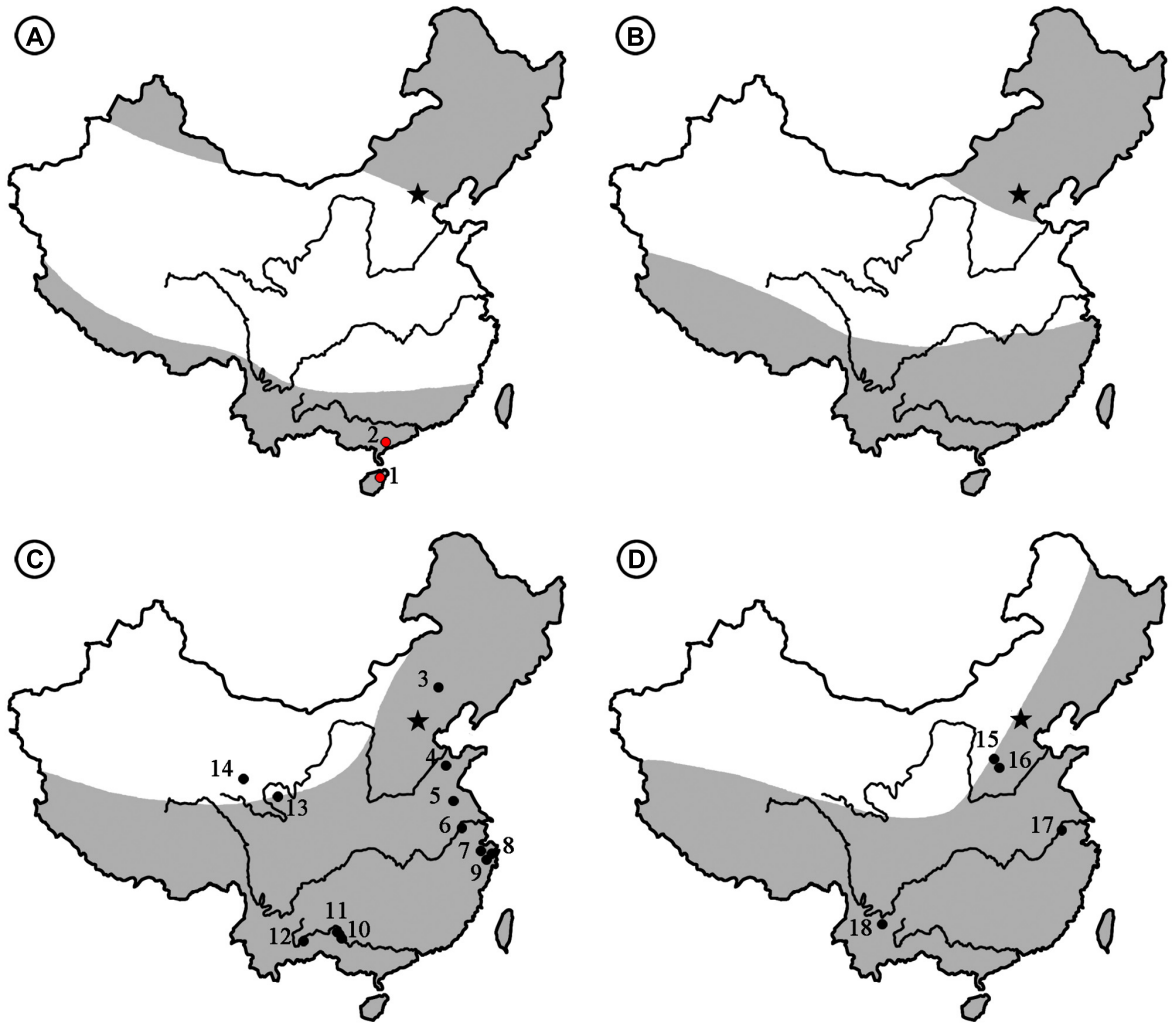
**The climate zones [revised from Sun and Wang (2005)] and distribution of *Podocarpium* megafossils from the Eocene to Pliocene in China. (A)** Eocene. (1) Changchang Basin, Hainan (the present paper). (2) Maoming Basin, Guangdong (the present paper). **(B)** Oligocene. **(C)** Miocene. (3) Chifeng, Inner Mongolia (Tao, 2000). (4) Linqu, Shandong (Hu and Chaney, 1940; Wang et al., 2007). (5) Sihong, Jiangsu (Li and Guo, 1982). (6) Nanjing, Jiangsu (Li and Guo, 1982). (7) Shengzhou, Zhejiang (Liu et al., 1996). (8) Ninghai, Zhejiang (Liu et al., 1996). (9) Tiantai, Zhejiang (Li, 2010). (10) Tiandong, Guangxi (Guo and Zhou, 1992). (11) Tianyang, Guangxi (Guo and Zhou, 1992). (12) Kaiyuan, Yunnan (Zhou, 1985). (13) Zeku, Qinghai (Guo, 1980). (14) Wulan, Qinghai (Liu et al., 1996). **(D)** Pliocene. (15) Taigu, Shanxi (Tao, 2000). (16) Yushe, Shanxi (Tao, 2000). (17) Nanjing, Jiangsu (Li and Guo, 1982). (18) Yuanmou Basin, Yunnan (Liu et al., 2002). Arid area is shown in white and humid areas is shown in gray.

Photographs of megafossils and cuticles (**Figures 2–4**) were adjusted and arranged using Adobe Photoshop 5.0 (San Jose, CA, USA) programs. A map for the fossil localites (**Figure 1**) was drawn using DIVA-GIS (version 7.5) software (LizardTech, Seattle, WA, USA) and modified by Adobe Photoshop 5.0. The distribution of fossil records of *Podocarpium* in the world map (**Figure 5**) and climate zones of different geological ages (**Figure 6**) were modified from the literature Song et al. (1983), Liu et al. (2001b), Sun and Wang (2005) and Wang et al. (2007). Terminology used in the specimen description follows Herendeen (1990), Gunn (1991), and the Leaf Architecture Working Group (LAWG) (1999). All specimens are deposited in the Museum of Biology, Sun Yat-sen University, Guangzhou, China.

## Results

### Systematics

Family Leguminosae Jussieu

Subfamily Caesalpinioideae DC.

Genus *Podocarpium* A. Braun ex Stizenberger

Species *Podocarpium eocenicum* Xu et Jin sp. nov.

### Specific Diagnosis

Fruit elliptical, ovate, or obovate, straight or slightly curved, not twisted; margins neither constricted nor winged; apex acute or obtuse, base acute, attenuate, or broadly cuneate, oblique slightly or obviously; stipitate, stipe length shorter than valve length, base of stipe prominent. Valves indehiscent or tardily dehiscent, with an invisible seed chamber. Epicarp dull, glabrous, with obliquely reticulate venations or cracked. Single seeded; seed length is oblique or parallel to the fruit lengths. Hilum apical or subapical. Funiculus short, thick and straight. Epidermal cells irregular tetragonal, pentagonal, or hexagonal with thickened anticlinal walls and smooth periclinal walls; stomatal complexes anomocytic.

### Holotype

CC254a, b (**Figures 2A,B**) (designated here; part and counterpart specimens; collected from Changchang Fm. of Changchang Basin in Jiazi Town, Hainan Island, China).

### Paratypes

CC256a, b (**Figures 3A,B**); CC698a (**Figure 3I**); CC1100a, b (**Figures 2F,G**); CC1162b (**Figure 2D**); CC1163a (**Figure 3F**); CC1164 (**Figure 2I**); CC1168 (**Figure 3C**); CC1171 (**Figure 3K**); CC1184 (**Figure 3H**); CC1188a (**Figure 3G**); CC1216a (**Figure 2E**); CC1217 (**Figure 2H**); CC1223 (**Figure 3J**); CC1297a (**Figure 3E**); CC1298 (**Figure 2C**); and CC1300 (**Figure 3D**) (designated here; collected from Changchang Fm. of Changchang Basin in Jiazi Town, Hainan Island, China). MMJ1–002 (**Figure 2J**) and MMJ1–003a (**Figure 2K**) (collected from Youganwo Fm. of Maoming Basin near Jintang Town, Guangdong, South China).

### Other Specimens Studied

CC255a, b; CC259a, b; CC597; CC698b; CC1101; CC1162a; CC1163b; CC1172a, b; CC1173–CC1176; CC1178; CC1179a, b; CC1183; CC1188b; CC1189a, b; CC1190a, b; CC1211–CC1213; CC1215; CC1216b; CC1218; CC1219a, b; CC1220–CC1222; CC1224a, b; CC1297b; CC1299; CC1301 (collected from Changchang Fm. of Changchang Basin in Jiazi Town, Hainan Island). MMJ1–003b (collected from Youganwo Fm. of Maoming Basin near Jintang Town, Guangdong, South China).

### Locality

Jiazi Town, Qiongshan City, Hainan Island, China; Jintang Town, Maoming City, Guangdong Province, China.

### Stratigraphic Horizon

Changchang Fm., middle Eocene; Youganwo Fm., middle Eocene.

### Repository

The Museum of Biology, Sun Yat-sen University, Guangzhou, China.

### Etymology

The specific epithet is based on its geological time, which notes that the fossils were collected in Eocene strata from the Changchang Basin and Maoming Basin, South China.

### Description

The fruits are 1.2–4.3 cm long by 0.6–1.6 cm wide, straight or slightly curved, not twisted; elliptical, ovate, or obovate shaped; stipitate. Margins are wingless and not constricted. Apex of fruit is acute or obtuse, while its base is acute, attenuate, or broadly cuneate, oblique slightly or obviously. The stipe is straight (**Figures 2D,E,H** and **3A–E,G**) or slightly curved (**Figures 2A–C,J** and **3H,K**), with a preserved length of 0.2–2.3 cm. Base of fruit stipe is prominent (**Figures 2A,B**). The valves are tardily dehiscent (**Figures 2A–C,I** and **3J,K**) or indehiscent (**Figures 2D–H,J,K**), 1.2–3.3 cm long by 0.6–1.6 cm wide. Externally the seed chamber is invisible. The epicarp is dull and glabrous, bearing numerous clear or less clear reticulate striations (**Figures 2G–I** and **3A,B,E,G,K**) on the surface oblique or slightly oblique to the length of the pod. Primary veins are obviously prominent, raised from one of the two sutures, and obliquely and subparallel, extending to other suture. Fine venations are gradually differentiated among primary veins. The pod splits along two sutures, and the suture is clearly prominent. Marginal placentation (**Figures 3A–H**) and seed is attached closely to apex of the ventral suture. Single seed is preserved in those valves that have not yet dehisced (**Figures 3A–I**). Seed is symmetrical and compressed, 0.5–1.7 cm long by 0.3–1.0 cm wide, and has an apical or subapical hilum with a short (ca. 2 mm), thick and straight funiculus on the top (**Figures 3A,B,E,F**). In the juvenile phase (**Figures 3A–E**), immature seed is near obovate, rounded in the apex, located near the apex of the ventral suture, the seed length is oblique or parallel to fruit length. It is oblong to ellipsoidal and swells in the center of the pod in the maturing phase (**Figures 3F–I**), and the seed length in relation to fruit length is oblique or parallel, and then falls out from the valve after it matures (**Figures 2A–C,I** and **3J,K**).

Epidermal cells from the outside of valves are irregular in shape, size, and arrangement (**Figures 4A,C–E,J,L**). Some cells are rectangular, variously elongated with straight anticlinal walls, while others are irregular tetragonal, pentagonal, or hexagonal with rounded or slightly undulate anticlinal walls. Anticlinal walls are not evenly thickened, and periclinal walls are smooth with one to multiple elliptical or round crystals (**Figure 4J**) in some cells observed by both LM and SEM. Cells are about 13–34 μm (average 19.4–24.6 μm) long and 8–22 μm (average 11.3–16.6 μm) wide. Stomatal complexes irregularly scattered over the cuticle are anomocytic (**Figures 4A,G–M**), with two guard cells surrounded by five or more subsidiary cells, about 46–84 μm (average 60.8 μm) long and 43–62 μm (average 50.2 μm) wide and the stomatal aperture is ca. 35 μm long (**Figure 4G**). The guard cells with conspicuous outer and inner stomatal ledges (**Figures 4G–M**) are smaller than subsidiary cells. No obvious trichome bases were observed on the cuticle. Epidermal cells from the inner side of valves (**Figures 4B,F**) are also irregular arranged, cells are tetragonal, pentagonal, or hexagonal with numerous crystals in some cells.

### Comparison

As the distinguishing characters of Leguminosae are described: the fruit is composed of a single carpel with a single row of seeds along one suture and dehiscent along two sutures (Herendeen, 1992a). The compressed fossil fruits described above apparently possess these features, and are assigned unequivocally to the fossil genus *Podocarpium* after detailed comparison with the previously reported records [Working Group of Cenozoic Plants of China (WGCPC), 1978; Gregor and Hantke, 1980; Herendeen, 1992a; Wang, 2006; Wang et al., 2007].

Specimens collected in the Changchang Basin of Hainan Island and Maoming Basin of Guangdong, South China, preserve relatively diverse morphologies with continuous changing cuticular structures, as described above. These morphological differences may occur because of natural intraspecific variation. Drawing on the experience of intraspecific variation in samara morphology of *Acer* and its implication in taxonomical studies of fossil *Acer* (Huang et al., 2013), investigations of morphological disparities of the same extant species throw much fresh light on the identification of fossil species. Since morphological variations, as we observed, are also present in the extant genera of Leguminosae, even in the same branch of one species, we are more inclined to group all these fossil pods into one species.

Single-seeded pods occur in a large number of unrelated genera in Leguminosae. Since it is difficult to distinguish those fruits which mostly bear similar morphological characters, Herendeen (1990, 1992a) concluded that features such as position of placentation, patterns of valve venation can be very helpful in identification. Although some specimens collected in South China are incomplete, especially the absence of stipe, clear venation structures (**Figures 2F–I** and **3A,B,E,G,K**) are shown on the surface of pods. Attenuated fruit margins and inconspicuous sutures are features of indehiscent pods, while fossil pods that were elastically dehiscent have prominent oblique striations on the valves (Herendeen, 1990). These subparallel oblique striations indicate a certain extent of lignification, which plays an important part in pod dehiscence and seed dispersal (Herendeen, 1990; Liu et al., 2001b). With regard to our specimens in the present paper, some pods have already dehisced, while other pods may not have done so yet, suggesting that these pods most probably have been preserved at different developmental stages; as a consequence, it would be beneficial to observe the position of placentation and changes that happened during the seed ontogenetic process. When in its juvenile phase, immature seed is near obovate with a short funiculus on the top, situated in the upper part of the pod and closer to the placental suture. It becomes bigger, elliptical or ovate, gradually situated almost in the middle of the pod and slightly oblique to the placental suture in its mature or approximate maturity phase. The short funiculus is still presented on the top of the seed. There are also some pods without seeds, suggesting that it may have been aborted (Bawa and Webb, 1984), or have already dispersed as was the case of several pods preserved in their dehiscent state.

In China, *Podocarpium podocarpum* was first reported from the Miocene of Shanwang Fm., Shandong Province (Hu and Chaney, 1940), and subsequently from other numerous localities [Working Group of Cenozoic Plants of China (WGCPC), 1978; Guo, 1980; Li et al., 1987; Guo and Zhou, 1992; Sun, 1999; Tao, 2000]. These fossils are frequently identified as *Podogonium oehningense* (Koenig) Kirchh. [Working Group of Cenozoic Plants of China (WGCPC), 1978; Sun, 1999] or *Podogonium knorrii* (Braun) Heer (Hu and Chaney, 1940). All those specimens reported from China were assigned to the same taxon *Podocarpium podocarpum* by Wang (2006) and Wang et al. (2007), although comparisons between Chinese and European *Podocarpium* reveal a few morphological differences (Wang et al., 2007). However, Wang (2006) was not sure about the monospecific treatment of the genus. Features of the fruits of *P. podocarpum* summarized from Herendeen (1992a) and Wang et al. (2007) are as follows: (1) The fruit dehiscent to tardily dehiscent, or indehiscent, single seeded, with a straight or slight curved stipe which is at least 2–4.1 cm long and about 1 mm wide; (2) the valves is elliptical, 1.5–2.9 cm long by 0.6–1.1 cm wide, apex is acute, base is acute or attenuate, slightly oblique, margins is not winged, and valve venation is either not observed or indistinct; (3) the placentation is near apex of the fruit; (4) the seeds are oblong, 12–15 mm long by 8–10 mm wide. *Podocarpium eocenicum* resembles this fossil species in general appearance, valve dehiscence, wingless and not constricted margin, placental position and single seed. However, the differences between these two species (**Table 1**) are mainly: (1) *P. eocenicum* has clear obliquely or slightly obliquely reticulate venations while *P. podocarpum* is either indistinct or not observed; (2) the ratio of stipe length to valve length is less than 1 for *P. eocenicum* while greater than 1 for *P. podocarpum*. In addition, the features from funiculus and hilum can be clearly observed in *P. eocenicum* while unknown in *P. podocarpum.* We also obtained intact epidermis of the new speices from the compressed pods which preserved at different developmental stages. Although some epidermal structures of *Podocarpium* have been described (Rüﬄe, 1963; Bůžek, 1971), those characters are all summarized from leaf remians and the structure of stomatal complexes is poorly presented. There are no data sources available to show the cuticular characters from the pods before our report. Detailed cuticular features of this new species *P. eocenicum*, especially the stomata structure, make a perfect complement to this genus.

**Table 1:** The differences between *Podocarpium eocenicum* sp. nov. and *P. podocarpum* (A. Braun) Herendeen from Europe and China (Heer, 1857; Herendeen, 1992a; Wang et al., 2007).

Species	Fruit				Seed				Localities	Age
	Valve size (cm)	Stipe/Valve ratio	Venation	Epidermis	Size (mm)	Seed position (to fruit length)	Funiculus	Hilum position (to seed length)		
*P. eocenicum* sp. nov. *P. podocarpum*	1.2–3.3^∗^ 0.6–1.6 1.5–2.9^∗^ 0.6–1.1	<1 >1	Obliquely Reticulate indistinct or not observed	Cells irregular tetragonal, pentagonal, or hexagonal; stomatal complexes anomocytic unknown	5–17^∗^ 3–10 10–15^∗^ 6–10	Oblique or parallel unknown	Up to 2 mm; thick, straight unknown	Apical or subapical unknown	South China Europe, NE and SW China	Middle Eocene Oligocene to Pliocene


Some palaeobotanists have insisted that there is a close relative relationship between *Podocarpium* and extant legumes, but the fossils do not comform to any single extant genus from both the fruit and leaflet morphologies (Heer, 1857; Herendeen, 1992a). Herendeen (1992a) considered that *Podocarpium* is similar to several genera in the tribes Detarieae DC. *sensu lato* (Caesalpinioideae) (here after referred to as Detarieae) and proposed four genera (*Gilletiodendron* Vermoesen, *Tessmannia* Harms, *Brachystegia* Bentham, and *Cryptosepalum* Bentham) restricted to tropical and subtropical Africa and some species of the pantropical genus *Cynometra* L. occurred only in Africa (e.g., *C. hankei* Harms, *C. leonensis* Hutch. and Dalziel) are most similar to *Podocarpium*, with special respect to the leaflet morphology and venation. Taking advantage of the monograph of Gunn (1991) and the herbarium collections maintained in the United States National Herbarium of National Museum of Natural History, Smithsonian Institution, we examinated most of the genera in Caesalpinioideae focusing on the pods bearing single seed, with special reference to the fruit shape, stipe, margins (constriction, the presence of wing) and epicarp features (sheen, hairs, venation) and the seed shape and position, etc. We found that more than 20 genera are comparable to *Podocarpium*. Herein, we also hold the idea that it cannot be directly related to any extant genus of Leguminosae (Herendeen, 1992a; Wang et al., 2007), because these extant genera share one or several features of *Podocarpium*, for example: (1) the genera with strictly single-seeded pods are: *Burkea* Bentham, *Daniellia* J. J. Bennett, *Kingiodendron* Harms, *Labichea* Gaudichaud-Beaupre ex de Candolle; *Lebruniodendron* J. Léonard; *Peltogyne* Vogel; *Prioria* Grisebach, *Stuhlmannia* Taubert, *Umtiza* Sim and *Vouacapoua* Aublet; (2) the genera with a long stipitate pods are: *Gleditsia* L., *Griffonia* Baillon and *Macrolobium* Schreber; (3) the genera with elliptical, ovate or obovate shaped pods are: *Arcoa* Urban, *Brenierea* Humbert, *Copaifera* L., *Crudia* Schreber, *Gilletiodendron* Vermoesen, *Guibourtia* J. J. Bennett, *Pseudosindora* Symington, *Stahlia* Bello, *Tessmannia* Harms and *Zenkerella* Taubert, etc.; (4) the genera with transverse or oblique reticulate venations are: *Burkea*, *Crudia*, *Gleditsia*, *Goniorrhachis* Taubert, *Guibourtia*, *Lebruniodendron*, and *Sindoropsis* J. Léonard. However, there is no one genus can match with all the features of the fruit and seed of *Podocarpium*.

It is noteworthy that the taxonomy of *Podocarpium* revised by Gregor and Hantke (1980), which focused on fruit morphology, was assigned it to the extant genus *Gleditsia*, especially related to the single-seeded fruits of *G. aquatic* Marshall and *G. heterophylla* Bunge. But this assignment was disproved because several important features of *Podocarpium* are inconsistent with *Gleditsia*: (1) Stipitate, single-seeded fruits similar to *Podocarpium* occur in many other legume genera; (2) the angle formed at the junction of the stipe and valves, as well as the fruit apex shape, is also confirmed to be a useless character because the fruits of many extant genera possess similar ranges of varitation in this structural feature (Herendeen, 1992a); (3) the position of placentation in *Gleditsia* is presented in the center of the pod, whereas in *Podocarpium* it is situated near the fruit apex; (4) the leaf characters clearly demonstrate that *Podocarpium* is different from *Gleditsia* (Herendeen, 1992a). Moreover, Liu et al. (2001b) also thought the association between *Podocarpium* and *Gleditsia* is unreliable after they re-evaluated the associated flowers with *in situ* pollen grains of *Podocarpium*. Thus, *Gleditsia* can be excluded from having a close relationship with the previous records and the specimens discussed in this paper.

## Discussion

It was once thought that legumes probably evolved in the humid tropics in the late Cretaceous (Sprent, 2007), and the remains (including pollen, leaflet, and fossil wood) of this age have been reported from many localities, such as the Caucasus, Sudan, Somalia and Mexico, and Siberia, Cananda, and Colombia, Central India, and China (Raven and Polhill, 1981; Muller, 1984; Giraud and Lejal-Nicol, 1989; Awasthi, 1992; Guo and Zhou, 1992; Herendeen et al., 1992; Shakryl, 1992; Wang et al., 2007). But many early records are unreliable and need reevaluation. The oldest currently recognized fossil of legumes appears during the early Paleocene (Giraud and Lejal-Nicol, 1989; Brea et al., 2008). Recently, according to molecular data and unequivocal legume fossil evidence, Lavin et al. (2005) fixed the family stem clade at 60 Mya, and estimated the age of the Leguminosae crown node at 59 Mya. They also noted that the oldest caesalpinioid, mimosoid and papilinoid clades were present from about 39 to 59 Mya. The fossil record documents that extensive diversification had taken place by the middle Eocene (Herendeen et al., 1992). A combination of the fossil record and extant geographic centers of legumes tribes, makes it evidently that the greatest legume diversity is concentrated in tropical America and Africa/Madagascar (Herendeen et al., 1992).

*Podocarpium* is one of the most common extinct genera of Leguminosae. Before our study, the earliest and most reliable record of this genus, named “*Gleditsia knorrii* Barbu” (Barbu, 1936; Gregor, 1985; Liu et al., 2001b), was reported from the early Oligocene of Romania and France. The pollen grains of *Fupingopollenites* from the early Eocene of eastern China (Zhang and Qian, 1992) were considered to be the oldest record of *Podocarpium* (Liu et al., 2001b). But recent palynological reports demonstrate that *Fupingopollenites* may be a representative of Verbenaceae (Song et al., 1999) or another dicotyledonous plant now extinct (Song et al., 2004; Wang and Harley, 2004). Therefore, whether it is reliable to determine the presence of *Podocarpium* by relying on palynological evidence is still questionable. The fossil species *Leguminocarpon* sp. (Erdei and Rákosi, 2009) recorded from the middle Eocene Csordakút (North Hungary) share some characters with *Podocarpium*, but it was eventually assigned to the genus *Leguminocarpon* due to the shortage of information. Although fruit fossil *Leguminocarpon lakhanpalii* Srivastava and Mehrotra (Srivastava and Mehrotra, 2010) from the late Oligocene of Assam shows close resemblance to *Podocarpium*, it differs from the latter in having a short thick single septum, and no cuticle of this species has been reported. So we are uncertain of its specific relationship to *Podocarpium*. *Podocarpium* was once reported in the Tertiary North America (Lesquereux, 1878; Berry, 1909; Brown, 1934), but these records were considered unreliable (Liu et al., 2001b). Therefore, our specimens collected from the middle Eocene of South China provide the definitive earliest evidence of this genus.

*Podocarpium* was supposed to have relationships with some genera in the Detarieae by Herendeen (1992a). So one possible opition of origin is Pan et al.’s (2010) suggestion that the more likely dispersal possibility of Detarieae would have been from south to north, and he deemed that the dispersal into Africa from Europe (Schrire et al., 2005a,b) is problematic because the fossil record of this group is much older in Africa than Eurasia. However, we didn’t find any record of *Podocarpium* repored from Africa or India. Alternately, Liu et al. (2001b) and Wang et al. (2007) proposed that this genus may have been originated in eastern Asia. According to the spatio-temporal distribution of this genus so far, of which almost all are reported from Eurasia (**Figure 5**), we speculate that *Podocarpium* had distributed in the South China at least in the middle Eocene, and then dispersed among Eurasia.

*Podocarpium* was probably a thermophilous, moisture-loving plant (Rüﬄe, 1963; Li et al., 1987; Liu et al., 2001b; Wang et al., 2007) and may have been an element of gallery forests (Herendeen, 1992a; Liu et al., 2001b; Wang et al., 2007). This speculation is exemplified by: (1) The middle Miocene Noroshi Flora from Noto Peninsula, Japan deposit containing leaves and pods of *Podocarpium* indicated a lagoonal environment and represented by a mixed mesophytic forest type. The climate of this flora was probably a little warmer and wetter than the present west end of Inland Sea, Japan (Ishida, 1970); (2) The localities of this genus reported from China before turned out to have had warm temperate-subtropical and tropical climates, such as the Miocene Shangwang Fm. in Shandong (Sun et al., 2002; Liang et al., 2003; Yang et al., 2007), or a temperate to warm temperate and arid climatic condition (Guo, 1980); (3) The habitat of *Podocarpium* in southern Germany is wet and warm. For example, the middle Miocene Schrotzbrug flora most likely represented a riparian forest vegetation (Hantke, 1954; Uhl et al., 2003), and the early/middle Miocene Radecker Marr flora was considered to be a lacustrine system with subhumid sclerophyllous forests or mixed mesophytic forests (Rasser et al., 2013). According to the plant assemblages, together with the palynological data, derived from the middle Eocene coal-bearing series of Changchang Basin (Yao et al., 2009; Spicer et al., 2014) and Maoming Basin (Aleksandrova et al., 2012, 2015) where *P. eocenicum* sp. nov. was collected, the climate of these two localities is warm and humid, very likely, the preferred environment of *Podocarpium*.

As generally understood, the Eocene climate was comparatively warm, i.e., warmer than any other period of the Cenozoic (Huber and Caballero, 2011; Quan et al., 2012). At that time the northward moving India Plate and the elements of the Eurasian continent had not yet merged (Chen et al., 1999). Song et al. (1983) divided the Eocene climate of China into three zones based on the palynofloral assemblages (**Figure 6A**). The broad arid-semiarid zone in the middle part of China became an important limiting factor of the eastern Asia flora in the Palaeogene (Guo, 1985; Tiffney and Manchester, 2001), but it had little influence on the development of *Podocarpium* in South China because it was part of the humid tropical to subtropical climate zone (**Figure 6A**), and it spread southwestward after its appearance here. During the Oligocene, the Turgai Straits separating Europe from Asia gradually closed (Liu et al., 2001a; Sun and Li, 2003) and the India Plate finally joined with Eurasia (Awasthi, 1992). This genus was able to enter Europe and reached areas such as Romania and France as fossil assemblages have recorded, but the persistence of the widespread arid band throughout the Oligocene (**Figure 6B**) (Song et al., 1983; Sun and Wang, 2005) made it difficult for *Podocarpium* to disperse northward in China. Up to now, *Podocarpium* does not have any record in the Oligocene of China. Even the whole legume family was less well documented in this period of time in China. In the Miocene, the climatic conditions of middle latitude Eurasia were generally favorable because the arid band disappeared (Sun and Wang, 2005; Wang et al., 2007). *Podocarpium* spreads extensively across subtropical and warm temperate areas of China, it goes northwardly to Zhejiang, Jiangsu, Shandong, Inner Mongolia, and Qinghai provinces, and southwestwardly to Guangxi and Yunnan provinces (**Figure 6C**). Meanwhile, *Podocarpium* occurs in large numbers in other parts of Eurasia, such as Germany, Switzerland, Italy, Romania, Poland, Czech, Hungary, Moldavia, Yugoslavia (Liu et al., 2001b), Austria, Japan (Uemura and Li, 2006; Wang et al., 2007). Due to the influence of the recently uplifted Tibetan plateau and subsequent climatic deterioration, the aridity of the Eurasian interior became more pronounced (Chen et al., 1999; Liu et al., 2001a; Sun and Li, 2003; Liu and Dong, 2013) and the distribution area of *Podocarpium* rapidly shrank. The megafossil records of this genus were found in the Pliocene floras of Yunnan, Jiangsu, and Shanxi provinces of China (**Figure 6D**) (Li et al., 1987; Tao, 2000; Liu et al., 2002). *Podocarpium* became extinct most likely in Eurasia after the Pliocene (Herendeen, 1992a; Liu et al., 2001b).

## Author Contributions

JJ and QX participated in the design of the study. JQ, JJ, and QX photographed specimens and arranged the figures. JQ and QX carried out the cuticle experiments and data analyses. QX, JJ, and ZZ conducted taxonomic treatments, evolutionary and phytogeographic interpretations. QX wrote the manuscript and formatted the text. All authors read and approved the final manuscript.

## Conflict of Interest Statement

The authors declare that the research was conducted in the absence of any commercial or financial relationships that could be construed as a potential conflict of interest.
