# Corrigendum: Eocene *Podocarpium* (Leguminosae) from South China and its biogeographic implications

**DOI:** 10.3389/fpls.2015.01036

**Published:** 2015-11-17

**Authors:** Qingqing Xu, Jue Qiu, Jianhua Jin, Zhekun Zhou

**Affiliations:** ^1^State Key Laboratory of Biocontrol and Guangdong Provincial Key Laboratory of Plant Resources, School of Life Sciences, Sun Yat-sen UniversityGuangzhou, China; ^2^Department of Paleobiology, National Museum of Natural History, Smithsonian InstitutionWashington, DC, USA; ^3^Xishuangbanna Tropical Botanical Garden, Chinese Academy of SciencesMenglun, China

**Keywords:** Eocene, Leguminosae, *Podocarpium*, phytogeography, South China

Due to a misunderstanding, the authors' names were incorrectly provided in the published article as

Qingqing Q. Xu, Jue Qiu, Zhekun K. Zhou and Jianhua H. Jin

and in the citation as:

Xu QQ, Qiu J, Zhou ZK and Jin JH (2015).

The correct names are:

Qingqing Xu, Jue Qiu, Zhekun Zhou and Jianhua Jin

and the citation:

Xu Q, Qiu J, Zhou Z and Jin J (2015).

The original article has been updated.

## Conflict of interest statement

The authors declare that the research was conducted in the absence of any commercial or financial relationships that could be construed as a potential conflict of interest.

